# Rapid improvements and subsequent effects in major depressive disorder patients with somatic pain using rTMS combined with sertraline

**DOI:** 10.1038/s41598-023-44887-w

**Published:** 2023-10-20

**Authors:** Yuanfeng Sun, Fei Lei, Ke Zou, Zhong Zheng

**Affiliations:** Neurobiological Detection Center, West China Hospital, Sichuan University, Chengdu, China

**Keywords:** Psychology, Medical research, Neurology

## Abstract

This study aims to explore changes in depression and pain for major depressive disorder (MDD) patients with somatic pain after repetitive transcranial magnetic stimulation (rTMS) using the event-related potentials (ERPs) technique. Eighty MDD patients with somatic pain were randomly assigned to drug therapy (DT) and combined therapy (CT) groups. CT group underwent intermittent theta burst stimulation over the left dorsolateral prefrontal cortex (DLPFC) with 800 pulses and 1 Hz over the right DLPFC with 800 pulses, 5 times a week for 3 weeks. All patients were given sertraline at 50–100 mg per day. All subjects were evaluated at baseline and at weeks three and six of therapy using the Hamilton Rating Scale for Depression (HAMD), Hamilton Anxiety Scale (HAMA), and Numerical Rating Scales (NRS), and the latency and amplitude of P300 and mismatch negativity (MMN) were measured. There were no significant differences in all indices between groups at baseline. At 3 weeks, HAMD subscale scores of Cognitive Impairment and NRS scores were significantly lower in the CT group than in the DT group. At 6 weeks, NRS and HAMD total scores in the CT group decreased significantly in the CT group compared with the DT group, especially for anxiety and pain, and the MMN and P300 latencies and P300 amplitude showed greater improvements. Our findings highlight that rTMS in combination with antidepressants is a rapid method of symptom improvement in patients with somatic pain with MDD and is helpful for cognitive impairment and anxiety.

## Introduction

Pain is one of the most common symptoms in patients with major depressive disorder (MDD), being present in 65% of cases^1^. Pain is associated with longer and harder to treat illness and poor health-related quality of life outcomes in MDD patients^2,3^. Compared to MDD patients without pain, those who suffer from severe pain are less likely to achieve remission and partial response, patients with an early improvement in pain were more likely to achieve remission^4^.

Cognitive impairment is another common and often persistent symptom of MDD. Patients with pain and cognitive impairment are more challenging to treat and have longer treatment durations. Furthermore, cognitive dysfunction may persist even after the patient's depressive symptoms have significantly resolved^5^. Thus, cognitive impairment in MDD patients with pain is severe. Treating pain may lead to improved cognitive performance in patients with depression and reduced depression in patients with cognitive impairment^6^. However, a more effective way to treat depression, pain, and cognitive dysfunction is required for patients suffering from MDD with pain.

Repetitive transcranial magnetic stimulation (rTMS) is a technique involving noninvasive stimulation of the cerebral cortex. Multiple studies involving rTMS in MDD patients with pain have reported that TMS significantly relieves pain in patients with MDD^7,8^. Though most studies have targeted the motor cortex for pain management, several studies clearly showed significant analgesic effects following rTMS over the left dorsolateral prefrontal cortex (DLPFC^9,10^. During depressive episodes, low activity in the cognitive control network (CCN), which includes the dorsal anterior cingulate cortex (DAAC) and the DLPFC, has been found in resting functional connectivity in MDD patients^11^. Additionally, two studies found that rTMS applied over the DLPFC positively impacted cognitive function in healthy subjects and elders with mild cognitive impairment^12,13^. Attention and vigilance can also be improved with rTMS^14^. However, there is relatively little literature on treating cognitive function with rTMS in cases of MDD with pain.

Lately, event-related potentials (ERPs) with a temporal resolution of milliseconds have increasingly been used for cognitive function evaluation. The most frequently reported components of the ERP are P300 and mismatch negativity (MMN). The P300 latency reflects the time spent on cognitive processing of the stimulus and the P300 amplitude is related to the quantity of attentional resources allocated during the task and memory performance^15,16^. Prolonged latency of P300 is a characteristic of cognitive impairment in depression^17^, and P300 latency is an important biological indicator for evaluating the severity of MDD^18^. In addition, it has also been suggested that reduced P300 amplitude in depressed patients is an important indicator of neurocognitive dysfunction^19^. MMN likely represents an automatic cerebral process, pre-attentive cognitive operations, and primitive intelligence^20,21^. For patients suffering from chronic pain, a study indicated that pain significantly reduces MMN amplitude, but no significant changes were observed on P300^22^.

However, ERP changes after rTMS treatment in patients with MDD and pain remain unknown. To fill this gap in the literature, the current longitudinal study explored the effects of rTMS-combined antidepressant pharmacotherapy on depression, pain, and cognitive function in MDD patients with pain. Sertraline was chosen for drug therapy for its efficacy and good tolerance.

We hypothesized that compared with the drug therapy group (DT), the combination therapy (CT) group (drug therapy combined with rTMS) would show more rapid improvements in depression, pain, and cognitive function. In addition, compared with the DT group, the latency or amplitude of ERP was hypothesized to be significantly enhanced in the CT group.

## Results

### Characteristics of participants

In total, 37 patients in the DT group and 38 in the CT group completed the study with a 92.5% and 95.0% completion rate, respectively. Three cases in the DT group were dropped after 3 weeks for taking other drugs. In the CT group, two cases were lost during the follow-up––one due to work and another who believed that the treatment was ineffective and required cumbersome daily therapy at the hospital. However, the score of the emotion and pain we evaluated is decreasing. The data was only analyzed for the included patients (DT, n = 37; CT, n = 38).

At baseline, there were no significant differences in age, gender, the course of disease, Hamilton Rating Scale for Depression (HAMD) score, Hamilton Anxiety Scale (HAMA) score, Numerical Rating Scales (NRS) scores, P300 and MMN latencies of P300, or P300 amplitude between the DT and CT groups (*p* > 0.1). Baseline characteristics of the study population are shown in Table 1.

**Table 1:** Baseline characteristics of the study population.

	DT group (n = 37)	CT group (n = 38)	*P*
Female (%)	29 (78.4)	28 (73.7)	0.788
Age(years)	42.9 ± 10.5	43.9 ± 10.7	0.682
Course of disease(month)	4.0 ± 3.2	4.0 ± 3.7	0.934
HAMD	31.6 ± 8.3	32.4 ± 8.1	0.685
HAMA	24.1 ± 7.3	23.3 ± 6.4	0.631
NRS	7.0 ± 1.4	6.9 ± 1.3	0.744
MMN latency (ms)	221.2 ± 27.1	224.8 ± 28.2	0.181
P300 latency (ms)	394.8 ± 27.2	392.2 ± 28.5	0.697
P300 amplitude (uv)	2.7 ± 2.2	2.5 ± 3.5	0.680

Results are shown as n (%) for the X^2^ test and mean ± SD for the independent t-test (two-tailed). *DT* the drug therapy group, *CT* the drug and rTMS combined therapy group.

### Comparison of the scores of the DT and CT groups at baseline, 3 weeks and 6 weeks

At baseline, 3 weeks, and 6 weeks, repeated-measures ANOVAs, with time and group as the factors, was used to compare the HAMA, HAMD and NRS scores; the P300 and MMN latencies; and the amplitude of P300 between DT and CT groups. The main effect of time was significant for HAMD, HAMA, NRS, P300 amplitude, and the latencies of P300 and MMN (*p *< 0.001). A group-by-time interaction effect was observed on the HAMD, HAMA, NRS and the amplitude of P300 (*p *< 0.001) (see Table 2).

**Table 2:** Comparison of the scores of DT and CT groups at baseline, 3 weeks and 6 weeks.

		DT group	CT group	Time effect	Group effect	Time x group effect
HAMD	Baseline	31.6 ± 8.3	32.4 ± 8.1	164.12***	2.857	9.614***
	3 weeks	25.9 ± 7.6	22.6 ± 8.6			
	6 weeks	21.3 ± 7.9	15.4 ± 7.2			
HAMA	Baseline	24.1 ± 7.3	23.3 ± 6.4	159.372***	14.024***	25.297***
	3 weeks	19.7 ± 6.5	11.0 ± 7.8			
	6 weeks	14.1 ± 6.4	8.1 ± 6.9			
NRS	Baseline	7.0 ± 1.4	6.9 ± 1.3	187.496***	15.961	11.597***
	3 weeks	5.5 ± 1.4	3.8 ± 1.6			
	6 weeks	3.9 ± 1.4	2.6 ± 1.2			
MMN latency	Baseline	221.2 ± 27.1	224.8 ± 28.2	8.143***	1.298	2.299
	3 weeks	215.9 ± 20.0	209.6 ± 20.1			
	6 weeks	214.6 ± 21.2	203.3 ± 18.6			
P300 latency	Baseline	394.8 ± 27.2	392.2 ± 28.5	22.05***	2.194	2.289
	3 weeks	386.1 ± 21.0	379.6 ± 19.1			
	6 weeks	385.0 ± 22.2	372.8 ± 16.6			
P300 amplitude	Baseline	2.7 ± 2.2	2.5 ± 3.5	16.477***	1.697	11.249***
	3 weeks	2.8 ± 2.8	3.2 ± 2.6			
	6 weeks	3.0 ± 3.2	5.3 ± 3.3			

*HAMD* Hamilton Rating Scale for Depression, *HAMA* Hamilton Rating Scale for Anxiety, *NRS* Numerical Rating Scales, *MMN* mismatch negativity, *DT* the drug therapy group, *CT* the drug and rTMS combined therapy group.

**Figure 1:**
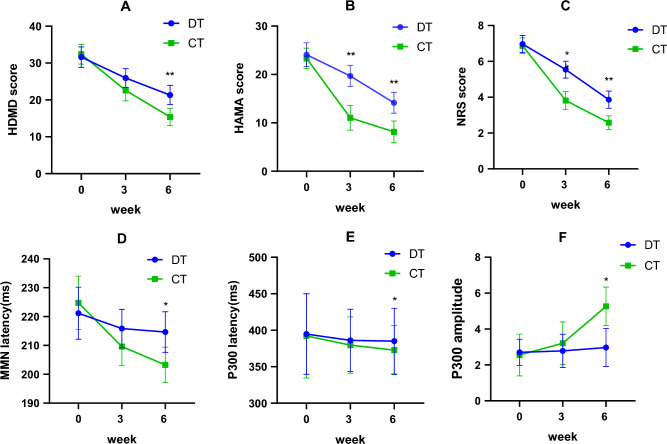
Mean HAMD (**A**), HAMA (**B**), NRS (**C**), the latency of the MMN (**D**) and P300 (**E**), and the amplitude of P300 (**F**) of the subjects in the efficacy analysis. Error bars indicate 95% CI of the mean. There were thirty-seven patients in the DT group and thirty-eight in the CT group. *HAMD* Hamilton Rating Scale for Depression, *HAMA* Hamilton Rating Scale for anxiety, *NRS* Numerical Rating Scales, *MMN* mismatch negativity, *DT* drug therapy group, *CT* drug therapy and rTMS combined treatment group. The difference between the CT and DT groups is displayed in the figure: *means *p* < 0.01, ** means *p* < 0.001.

Following observation of the interaction, analyses of variance were performed to identify the extent of change in each group. The HAMD, HAMA and NRS scores significantly differed between the DT and CT groups at 6 weeks (*p* < 0.001). HAMA (*p* < 0.001) and NRS (*p *< 0.01) scores showed significant differences at 3 weeks. The MMN latency, P300 latency and P300 amplitude were significantly different between the DT group and CT group at 6 weeks (*p* < 0.01) (See Fig. 1).

## Discussion

To our knowledge, this was the first study examining the primary changes in ERPs, improvements and effects of a combination treatment with rTMS and antidepressants on depression, pain and cognitive function in untreated MDD patients with somatic pain. Our findings highlighted that rTMS combined with antidepressants led to rapid symptom improvements in patients suffering from depression and pain.

The combined treatment group, which received high-frequency (50 Hz) iTBS stimulation and right 1 Hz low-frequency stimulation, showed greater improvements in pain at 3 weeks than the drug treatment group. Previous studies on pain and depression have shown that rTMS with frequencies greater than 5 Hz can significantly improve depression and pain symptoms in patients^23^. A meta-analysis suggested that current research mainly focuses on the 10–20 Hz frequency range, particularly 10 Hz, with only two reports in the literature regarding treatments at 20 Hz^8^. The current study implemented high-frequency (50 Hz) stimulation of iTBS, which has been proven to be the most effective treatment mode for depression, in addition to low-frequency rTMS. The pain network consists of the insula, cingulate cortex, and somatosensory cortices. There is an increase in activity in the dorsal anterior cingulate cortex (dACC) and the anterior insula, which the DLPFC regulates through the pain control network^24^. The DLPFC, specifically in the right hemisphere, has been suggested to play a role in pain, emotion, and social decision-making^24^. Recent literature has suggested that iTBS over the left DLPFC can improve muscle activation patterns during challenging postural control tasks, providing a new approach to treating chronic low back pain^25^. We further confirmed the role of this treatment method in pain treatment.

Additionally, using low-frequency rTMS in the right DLPFC has been shown to increase tolerance to human experimental pain^10^ and alleviate the impact of anxiety^26^. Therefore, the low-frequency 1 Hz stimulation in the right DLPFC in our study may also play an essential role in relieving patients' anxiety and pain. Thus, although medication can reduce pain in some patients with depression, combined TMS can improve physical symptoms more quickly.

Our study also demonstrated that the subscale score of cognitive impairment in the CT group was significantly lower than in the DT group, both at 3 weeks and 6 weeks. MMN and P300 latencies were significantly decreased and P300 amplitude dramatically increased in the CT group compared to the DT group at 6 weeks_,_ suggesting that rTMS had a positive effect on cognitive function. This accords with previous studies reporting that rTMS had a positive impact on cognitive function^12,13^. In our study, iTBS was applied to the left DLPFC and 1 Hz standard stimulation to the right DLPFC. A study using iTBS to stimulate the left DLPFC in healthy people suggested that iTBS stimulation could shorten the P300 latency^27^. In addition, in an investigation targeting depression patients, active rTMS was associated with an increase in P300 amplitude and decrease in HAMD score compared to sham rTMS^28^. These two studies further confirmed our research findings. However, a review^29^ found the conclusions regarding rTMS effects on brain stimulation and P300 latency and amplitude to be inconsistent. In three studies mentioned in the review that used high-frequency stimulation, one found a decrease in latency, another found an increase, and the third found an increase in P300 amplitude. These inconsistencies may be related to differences in the stimulation site, frequency and duration across studies. There are relatively few studies on the effects of rTMS on MMN. Currently, a study^30^ on occipital rTMS stimulation combined with escitalopram oxalate in patients with depression showed that rTMS can shorten the latency of MMN and reduce HAMD scores, further confirming that rTMS can improve the pre-attention processing ability and cognitive function of patients with depression. 40-Hz rTMS can prevent gray matter volume loss and enhance local functional integration within the bilateral angular gyrus, as well as global functional integration in the bilateral angular gyrus and the left middle frontal gyrus, which are related to effective improvements in cognitive function^31^. Previous evidence has shown that routine high-frequency rTMS promotes neurogenesis in the human motor cortex more effectively than in rat models^32^. iTBS has also been shown to have a significant role in reducing cognitive dysfunction after stroke^33^. These findings further confirm the role of iTBS in improving cognitive function and the role of rTMS in enhancing cognitive function in MDD, and may explain why the treatment effect and ERP changes were more significant in the CT group than the DT group in the present study.

More interestingly, the anxiety score and pain relief score showed a significant decrease after 3 weeks. However, there was a significant difference in depression score and ERP evaluation after 6 weeks. These results may indicate that rTMS improves anxiety and pain quickly, but that changes in cognitive function are relatively slow and only have a significant effect at 6 weeks. A multicenter depression study in the United States showed that rTMS can improve depression scores by the second week, and patients' depression scores can significantly improve by the sixth week^34^. A Canadian study on the treatment of generalized anxiety disorder showed that high-frequency rTMS stimulation can quickly alleviate anxiety and maintain a stable therapeutic effect after 1 month^35^. This aligns with our finding that rTMS can quickly alleviate depression and anxiety in patients. However, the changes in cognitive function were also significantly different in the combined therapy group compared to the drug treatment group at 6 weeks of treatment, indicating that TMS has more advantages to cognitive function changes than drug treatment. This is related to the regulation of neuroplasticity by rTMS. Cognitive function can be improved in mild cognitive impairment (MCI) and Alzheimer's disease (AD) by regulating neural plasticity by rTMS^36^. However, this plastic change requires a specific treatment cycle to achieve a more significant effect. Some studies have also suggested that rTMS can regulate the activity of dopamine and GABA neurons to improve memory^37^.

As systematic reviews have reported that active rTMS is significantly more effective than sham rTMS in response and remission rates^38^, sham rTMS was not applied in the control group. This is a limitation of the study which will be amended in future research. The location of the stimulation site in our study is not accurate enough, and future research should use MRI combined with fixed positioning to improve the accuracy of the treatment site.

## Conclusion

Our results highlight that therapy involving rTMS in combination with antidepressants is an effective method for rapid symptom improvement in MDD patients with somatic pain. The combination therapy was helpful for many secondary symptoms such as cognitive impairment, and anxiety.

## Methods

### Subjects

Eighty MDD patients with moderate somatic pain were recruited from the Mental Health Center and the Neurobiological Laboratory, Sichuan University, West China Hospital. They had not received any analgesics or anti-depressants for at least 2 weeks before enrollment. Patients were randomly assigned to the DT group (n = 40) and CT group (n = 40) according to a computer-generated list. An experienced psychiatrist blinded to the group assignment performed psychiatric examinations on all patients to diagnose MDD. Inclusion criteria were: aged 18–60 years, met DSM-V criteria for MDD, 24-item HAMD score > 20, NRS score ≥ 4. Patients with other Axis I and Axis II psychiatric disorders were excluded. Exclusion criteria also included treatment with electroconvulsive therapy (ECT) or rTMS in the last year, epilepsy, pregnancy and contraindications to magnetic exposure (e.g., cranial plates). All subjects were of the Han nationality, and were right-handed, with normal hearing and no severe or acute medical conditions, based on clinical evaluations and medical records.

All participants provided written informed consent before the start of the study. All methods were performed according to the relevant guidelines and regulations, which were approved by the Human Research Ethics Committee of West China Hospital, Sichuan University. Sertraline was given at a daily dose of 50 mg for 1 week, followed by a daily dose of 100 mg for both the DT and CT groups.

### rTMS stimulation and evaluations

rTMS simulations were carried out using a Magstim Rapid2 stimulator (Magstim Company, Spring Gardens, UK) with a figure-of-eight coil (double wings of 70-mm diameter). Coil placement was 5 cm anterior to the left and right motor hotspots. The resting motor threshold (RMT) of the right abductor pollicis brevis muscle was used to determine the lowest strength of TMS needed to elicit at least five motor-evoked potentials ≥ 50μV over 10 trials. Patients in the CT group received 15 sessions of bilateral active rTMS (five times a week for 3 weeks). The total pulse was 1600 per session with 90% RMT. First, intermittent theta burst stimulation (iTBS) was applied to the left DLPFC for 800 pulses, then, 800 pulses were applied to the right DLPFC at 1 Hz.

ERPs were measured with MEB-9200 electromyogram/evoked potential equipment (Neuropack, Nihon Koden, Japan). The recording electrode was placed at Cz in the 10/20 system and the reference electrode was placed as a parallel electrode at the bilateral mastoid. MMN was evoked via headphones in the auditory oddball paradigm. Standard stimuli (60 dB nHL, 1000 Hz tones, envelope line of rise-plateau-fall were 10–100-10 ms) and deviant stimuli (80 dB nHL, 2000 Hz tones, the same as the envelope line) were presented in a ratio of 80%:20% in a randomized sequence, with a stimulus ratio at 1 Hz. The signals were averaged 40 times. MMN was obtained using subtractive processing between signals induced by deviant and standard stimuli in the absence of active attention. Subjects were not instructed to give any response to the target, only to lie relaxed with their eyes closed. The P300 was recorded under the same odd-ball paradigm, 30 times overlaid, and the waves were digitally filtered with a bandpass of 0.1 − 100 Hz. Subjects were instructed to press a button in response to the target. Analysis time and sensitivity were 100 ms/div and 20 μV/div, respectively, for all data. For all subjects, ERPs were measured and HAMD and NRS scores were evaluated three times: at baseline, after 3 weeks, and after 6 weeks.

### Statistical analysis

Data preprocessing was performed using SPSS 22.0 software. The demographic material and experimental indices (HAMD and NRS scores, ERP data) were analyzed at baseline, 3 weeks, and 6 weeks. The gender ratio of the two groups was analyzed using the Pearson chi-square test. All tests were performed with two-sided significance levels (P < 0.05).

Initially, independent sample t-tests were used to compare the baseline characteristics of the DT and CT groups (Table 1). We then conducted a series of repeated-measures analysis of variance (ANOVA) for each outcome variable to assess differences across time (three and six weeks) and between study groups (DT and CT), as well as for interactions between time and study group. For these analyses, the assumptions of sphericity and homogeneity of the variances were tested using Mauchly and Levene tests. The Huynh–Feldt correction was applied if there was a violation to the sphericity assumption.

## Data Availability

The datasets used and analyzed during the current study are available from the corresponding author on reasonable request.
